# Hospital Consultants and Panel Practice

**Published:** 1913-12-13

**Authors:** 


### The Hospital

The Workers' Newspaper of Administrative Medicine and Institutional Life, Administration, National Insurance and Health.

No. 1433, Vol. LV. SATURDAY, DECEMBER 13, 1913. PRICE ONE PENNY.

### HOSPITAL CONSULTANTS AND PANEL PRACTICE.

The medical benefit sections of the Insurance Act and its Regulations, which for all material purposes at the present time mean the panel system, so directly and intimately concern the general practitioner, paneller or not, that there is a tendency to regard the consultant as one who is not affected at all by the Act and to envy him accordingly. It is, of course, true that consultants as a class are comparatively much less affected by National Insurance than are general practitioners; but it is not the fact that they can therefore afford to neglect it, to take no interest in it, or to hold aloof from current medical politics which centre almost exclusively round the Act and its consequences.

In the very large towns, especially where teaching hospitals exist, the visiting staffs of the voluntary hospitals are purely consultants; they work, often very hard, for many hours a week in the hospitals without any fees or salaries, and in so doing they must inevitably give of their skill and labour to enormous numbers of persons insured under the Act. As matters stand at present they neither get nor expect to get a single penny from the funds administered under the Act, although it is indisputable that but for the voluntary hospitals and their unpaid staffs the whole machinery of the Act would break down hopelessly. Still, a day may come, possibly sooner than most people imagine, when some form of State subsidy will be inevitable to save the voluntary hospitals from bankruptcy; and it is quite likely that in such an event the consultants as a class might claim salaries in return for their services. These considerations will serve to show that the financial administration of the Act and its subsidiary authorities, and the future position of the voluntary hospitals, are so inextricably bound together that in time to come they may both influence very materially the future of consulting practice and the prospects of the consultant class.

In county hospitals and institutions of similar standing there is usually no rule requiring the members of the visiting staffs to be pure consultants; and although some of the seniors sometimes are largely or wholly consultants, the great majority certainly are not. Whether such visiting medical officers should or should not be permitted to "go on the panel" (if they desire to) is a question which has already given rise to feuds and heart-burnings at institutions where attempts have been made to limit the private practice of medical officers in this sense. Broadly speaking, it is only a small minority of such visiting staffs that will be panellers, so that the question is not likely often to arise. But, since so many Insurance patients are sent to hospitals by panel practitioners, and those who treat these patients in the hospitals are professional neighbours of those who send them in, it is obvious that for the staffs also, even though not themselves on the panel, the Act, and especially its future ultimate effects, are matters of real importance. In charities of the cottage-hospital type, where the local practitioners of repute, panellers or not, are either on the staff or have the *entrée*, the panel principle is, if anything, even more brought home to them, for they have to treat their own insured patients as an act of charity in serious illness, because the Act only provides and pays for the treatment of minor illnesses and not of severe ones.

All classes of hospital visiting medical officers are, it will be seen, touched in some way or another by National Insurance. The strictly consultant class have not, as a body, everywhere quite realised that the panel agitation affects them much, because their contact with it is less direct than that of the medical officers of smaller and local hospitals. There is, however, one exceedingly important aspect of the non-panel movement to which the consultants will do well to pay heed. A large percentage of their patients come to them on the recommendation of a private practitioner. Private practitioners are to some extent already divided into two great groups since "medical benefit" came into being—panellers and non-panellers—and the tendency of the gulf is to widen. Beyond all doubt the *clientèle* of the non-panellers contains the vast bulk of the people who resort to, and pay for the advice of, consultant practitioners. It is plain, therefore, that of the two classes, the non-panel men are those with whom the worldly wise consultant will take care to remain on good terms. Running with the hare and hunting with the hounds is a difficult art, at which only a select few are adept. The majority will find it necessary to take one side or the other, and common prudence dictates which side it shall be. Hitherto there has been a tendency for the consultants to hold aloof from the struggle, but they must inevitably be drawn into it eventually, for reasons already given, and we think they will do well to ponder the situation carefully and immediately.

